# Discriminant spectrogram local descriptors for electrocardiography biometric authentication

**DOI:** 10.1371/journal.pone.0343293

**Published:** 2026-02-27

**Authors:** Haiying Liu, Yuxin Shang, Haiyan Lin

**Affiliations:** 1 School of Computer Science and Technology, Xinjiang University, Urumqi, China; 2 Joint International Research Laboratory of Silk Road Multilingual Cognitive Computing, Urumqi, China; 3 Xinjiang Multimodal Intelligent Processing and Information Security Engineering Technology Research Center, Urumqi, China; 4 School of Economics, Xinjiang University of Finance and Economics, Urumqi, China; 5 Computer Department, Xinjiang Qitai Secondary and Technical School, Qitai, China; Polytechnic University of Marche: Universita Politecnica delle Marche, ITALY

## Abstract

In recent years, Electrocardiogram (ECG) biometric authentication has emerged as a hot topic in biometrics research due to its unique advantages including intrinsic aliveness characteristics and convenience for users. However, due to the non-stationary and nonlinear nature of ECG signals, there are still some challenges to be addressed for the application of ECG biometric authentication. In this paper, we propose a method that employs the short-time fourier transform (STFT) and a local binary descriptors learning method for ECG biometric authentication. Specifically, we first convert ECG heartbeats into two dimensional spectrogram images by STFT. Second, we extract pixel differential vectors (PDVs) from each point in the spectrogram images of the training ECG heartbeats. Third, we learn a projection matrix to map these PDVs into low-dimensional binary descriptors with three objectives: 1) The error between the original PDV and binary descriptor is minimized. 2) The intra-class variation of the local binary features is minimized and the inter-class variation of the local binary features is maximized. 3) The *L*_2,1_ norm of the learned binary descriptors is minimized. Finally, we represent each spectrogram as a histogram feature by clustering and pooling these binary descriptors. Experiments on the database verify that the proposed method outperforms other existing ECG biometric authentication methods in terms of performance.

## 1 Introduction

Biometrics is the technology that makes intelligent systems to produce automatic identification by acquiring and analyzing distinctive physiological and behavioral characteristics from an individual. However, they have always meet several challenges: the presence of spoofing attacks, the required massive data, and the high cost of device for computing and storing. To overcome these challenge, a number of works have shown that the bioelectrical signals can use as biometrics traits, such as Electroencephalogram (EEG), Electromyography (EMG) and Electrocardiogram (ECG). Among them, ECG biometric authentication has attracted a lot of interest lately due to convenience collection, continuous accessibility, liveness detection, and internal features. In recent, ECG biometric authentication systems have been widely studied and applied in various fields of social security, such as authentication patient in medical system, continuous authentication for web service, and driver identification. The existing methods of ECG biometric authentication include fiducial methods, non-fiducial methods and deep learning based methods. Fiducial methods [1–3] extract amplitude, interval, angle, slope of the wave as features. The accuracy of these methods is dependent on accurately identifiable performance of the fiducial markers. However, it is difficult to detect the fiducial markers when noise and variability distort the ECG wave and make the measurements unreliable. In addition, these methods rely heavily on extensive feature engineering. To overcome these limitation, non-fiducial methods have been presented. These methods segment the ECG signal into windows, and extract features directly from these windows segments, such as wavelet coefficients [4], sparse representation [5] and ECG signal power spectral density [6]. However, the key drawback of non-fiducial methods lies in their high computational cost [7]. In contrast, deep learning based methods, which can automatically generate features, have been widely adopted for ECG biometric authentication [8–10], and the excellent performance has been achieved. Nevertheless, deep learning based methods require large datasets, along with substantial computational resources and training time during the model training stage [11].

In this paper, we aim to learn discriminative and robust binary descriptors for the spectrograms of ECG heartbeats to enhance the representation power of local features. Our motivations are two-fold. First, the spectrogram, as a temporal and frequency domain representation of ECG signals, has demonstrated remarkable performance in various ECG signal tasks, including ECG stress classification [12], ECG heartbeat classification [13] and ECG biometric authentication [14]. This inspires us to explore the time-frequency information from ECG signal for ECG biometric authentication. The spectrogram is generated from an ECG heartbeat based on the idea of dividing the ECG heartbeat into segments and then employing the fast fourier transform (FFT) to each segment. It can better capture the spectral information in ECG heartbeat at different temporal intervals. Several existing studies [11–15] have transformed the ECG signal into spectrogram images and achieved high performance. Second, local feature learning methods have yielded impressive results in numerous vision tasks, such as face recognition [16], finger vein recognition [17], palmprint recognition [18] and few-shot image classification [19,20]. These works motivate us to design a novel local feature learning method for ECG spectrogram images.

Based on the above motivations, we have developed a discriminant spectrogram local descriptors (DSLD) for ECG biometric authentication. First, DSLD extracts pixel differential vector (PDV) from the spectrograms of ECG heartbeats and learn a project matrix to map each PDV into a binary descriptor. Since values from different radius are simply concatenated into a PDV, the semantic information cannot be fully exploited. To address this issue, we ensure that PDVs from images of different classes are separated and those from images of same class are clustered together. Additionally, we impose an *L*_21_ norm constraint on the project matrix to enable the selection of discriminative features. Then, we learn a codebook from all binary descriptors by *k*-means method and construct a histogram representation for each ECG heartbeat.

## 2 Background

In this section, we will briefly introduce the necessary background for this work, such as ECG biometric authentication methods and linear discriminant analysis, which are related to our work.

### 2.1 ECG biometric authentication

The ECG biometric authentication can be divided into traditional machine learning based methods and deep learning based methods.

**Traditional machine learning based methods:** According the feature extraction process, traditional machine learning based methods can be subdivided into fiducial and non-fiducial methods.

Fiducial methods for ECG biometric authentication is based on ECG fiducial markers to extract features, which depends on the morphology of ECG and the time relationship between fiducial markers. Biel et al. [1] extracted 30 features relative to onset, duration and amplitude of P wave, T wave, R wave and QRS wave. Palaniappan et al. [21] extracted six features, such as R-R interval, R amplitude, QRS interval, QR amplitude, RS amplitude, and form factor of the QRS segment. Paiva et al. [2] extract three temporal distance between the Q, R, S and T wave,such as time intervals of QT, RT, and ST. Choi et al. [3] suggest eight fiducial features, such as amplitude of P wave, Q wave, R wave, S wave, and T wave, intervals of PQ, QS, and ST. While fiducial methods have satisfied identification requirements criteria, accurate detection of fiducial points remains a big challenge.

Non-fiducial methods for ECG biometric authentication do not require to localization fiducial points in ECG heartbeat and depend on thoroughly analyze the ECG signal in the time or frequency domain. Srivastva et al. [22] extracted autocorrelation coefficients of ECG signal, and then use the discrete cosine transform (DCT), discrete fourier transform (DFT), and walsh-hadamard transform (WHT) respectively, and it was found that recognition effect of the DFT was better. Dar et al. [4] extracted the wavelet coefficients by discrete wavelet transform, and adopted the nearest neighbor classifier for identification. Gutta et al. [23] use the discrete cosine transformation coefficient of the autocorrelation coefficients as initial feature, and simultaneously performed feature learning as well as classification training to improve the recognition performance. Wang et al. [24] extracted the multi-scale differential feature from ECG signals and adopted the matrix factorization for ECG-based identity recognition. Xu et al. [5] proposed a sparse representation by learning personalized dictionaries for ECG-based identity recognition. Huang et al. [25] proposed a framework of multi-feature spare representation for ECG identification. Fatimah et al. [26] extract the essential attributes of ECG heartbeat cycle by the fourier decomposition method (FDM) and phase transform (PT), and adopted the random forest, support vector machine and ensemble subspace discriminant (ESD) for ECG-based identity recognition. Biran et al. [6] adopted the dynamic changes of the QRS characteristics of ECG signals and the dynamic changes of ECG signal power spectral density for ECG-based identity recognition.

In recent years, deep learning has achieved relatively good results in the fields of images and natural language, and the field of ECG recognition has also begun to use deep learning technology to improve recognition performance.

**Deep learning based method:** Deep neural networks have powerful feature extraction capabilities, which can eliminate noise and redundancy, and more and more studies use deep neural network technology for ECG-based identity recognition. Luz et al. [8] input the spectrogram of ECG signals to the convolutional neural network (CNN) for feature extraction and achieved good results in the ECG biometric database collected multiple times in different time periods. Labati et al. [9] propose Deep-ECG method for ECG biometric authentication which uses convolutional neural network and generates a real-value template and a binary template for matching. Zhao et al. [10] blindly segmented the ECG signal, transformed the segmented ECG signals into image, and input images into CNN for discriminative feature learning. Abdeldayem et al. [27] transformed ECG signal fragments into spectral correlation images and fed it into CNN. Li et al. [14] extract features by first CNN and adopted second CNN for ECG biometric authentication. Salloum et al. [28] adopted recurrent neural network (RNN) for ECG biometric authentication. Zhu et al. [15] proposed dual-domain low-rank fusion deep metric leaning for ECG biometric authentication. Byeon et al. [29] ensemble of spectrograms, log-spectrograms, Mel-spectrograms, and MFCCs, and achieved good performance in the PTB ECG database. Srivastva et al. [30] input the cardiac images into pre-trained DenseNet and ResNet, and fused the two networks for good performance on the PTB and CYBHi ECG databases. Thentu et al. [31] performed continuous wavelet transform to obtain a multi-scale representation for the heartbeat, and adopted a pre-trained neural network by the imageNet dataset to achieve good performance. Kim et al. [32] proposed a bidirectional recurrent neural network (BRNN) based on long short-term memory (LSTM) for biometric task and classification. Caterina et al. [33] designed CNN for ECG biometric authentication for patients with heartbeat disease. Jyotishi et al. [34] designed a hierarchical long short-term memory (HLSTM) model to capture different abstract temporal variation, and used the attention mechanism to extract identity information from ECG complexes. Islam et al. [35] adopted CNN, LSTM and transformer for ECG biometric authentication, and achieve good result on MIT-BIH and ECG-ID database.

Deep learning shows good performance depended on large-scale training data for massive hyperparameters tuning. However, the databases of ECG biometric authentication are relatively small, making it difficult to adapt to a deep learning model.

### 2.2 Linear discriminant analysis

Linear discriminant analysis (LDA) is one of the most well-known methods to extract discriminative features for pattern classification. LDA utilizes the label information to learn a discriminant projection that can greatly enlarge the intra-class distance and reduce the inter-class distance so as to improve the classification accuracy. Assumption there are *c* classes, *n*_*i*_ is the number of samples of the *i*th class, $n=\sum_{i=1}^{c}n_{i}$ denots the total number of all samples, $x_{ij}\in R_{d}$ is the *j*-th sample of the *i*-th class. LDA obtain a discriminant projection by Fisher criterion as Eq (1),

$$W=\max_{W^{T}W=I}\frac{Tr(W^{T}S_{b}W)}{Tr(W^{T}S_{W}W)},$$ (1)

where *S*_*w*_ and *S*_*b*_ are the intra-class and inter-class scatter matrices, respectively. In particular, the definitions of *S*_*w*_ and *S*_*b*_ are given by Eqs (2) and (3).

$$S_{W}=\sum_{i=1}^{C}\sum_{j=1}^{n_{i}}(x_{ij}-m_{j})(x_{ij}-m_{j}{)}^{T},$$ (2)

$$S_{b}=\sum_{i=1}^{C}n_{i}(m_{i}-m)(m_{i}-m{)}^{T},$$ (3)

where $m_{i}=\frac{1}{n_{i}}\sum_{j=1}^{n_{i}}x_{ij}$ is the mean of samples of the *i*-th class, $m=\frac{1}{N}\sum_{i=1}^{C}\sum_{j=1}^{n_{i}}x_{ij}$ denotes the mean feature of all samples. Since problem in Eq (1) is difficult to solve, researchers convert it into the optimization problem as shown in Eq (4).

$$W=arg\min_{W^{T}W=I}Tr(W^{T}(S_{W}-\beta S_{b})W),$$ (4)

where *β* is a small positive constant. After solved the problem Eq (4), the projection can make the samples between classes separate and the samples within classes together. Therefore, many works widely use LDA in various applications. However, LDA is sensitive to noise and outliers, and fails to deal with data of non-Gaussian distribution.

## 3 Materials and methods

**Fig 1:**
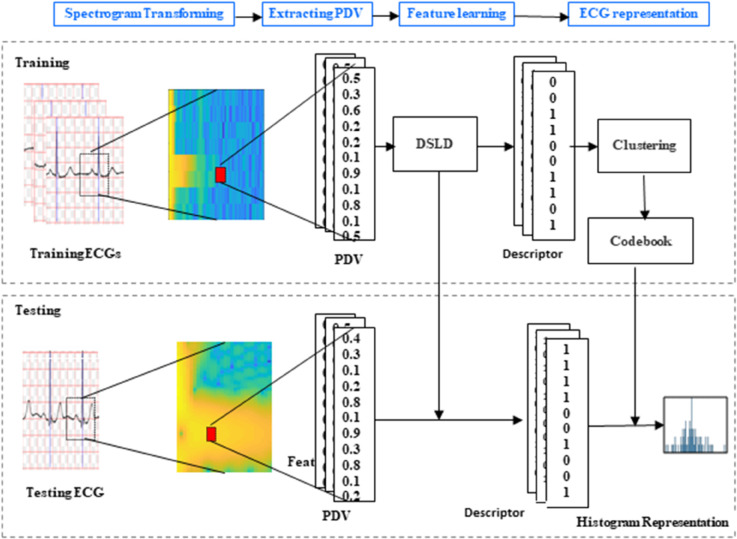
Architecture of discriminant spectrogram local descriptors learning and codebook clustering for ECG signal.

In this section, we elaborate on our proposed method. As depicted in Fig 1, the pipeline of our proposed method consists of five main stages.The first stage is preprocessing. In this phase the raw ECG signal is filtered using a bandpass filter in order to remove the noise, then it be segmented into fixed-length windows around each R-peak to ensure that each segment represents one QRS complex. The second stage involves two dimensional spectrogram conversion. We employ the STFT to extract the time-frequencies spectrogram for the ECG heartbeat. The third stage is PDV extraction, where a real value vector is computed for each pixel in the spectrogram. The fourth stage focuses on learning local binary descriptors. To extract discriminant descriptors, we use LDA combined with *L*_2,1_ regularizer. Finally, we leverage a bag-of-words framework to organize the learned local descriptors, thereby constructing a global representation for each ECG heartbeat.

### 3.1 Data preprocessing

ECG signal is one of the key factors in reducing the performance of biometric system, mainly including baseline drift (BD) noise, muscle artifacts (MA) noise, and power frequency (PF) noise. In this work, data preprocessing is same as our previous work [36], which involves signal denoising, heartbeat segmentation, and outlier removal. Interested readers can refer to the work [36].

### 3.2 Two dimensional spectrogram conversion of ECG signal

**Fig 2:**
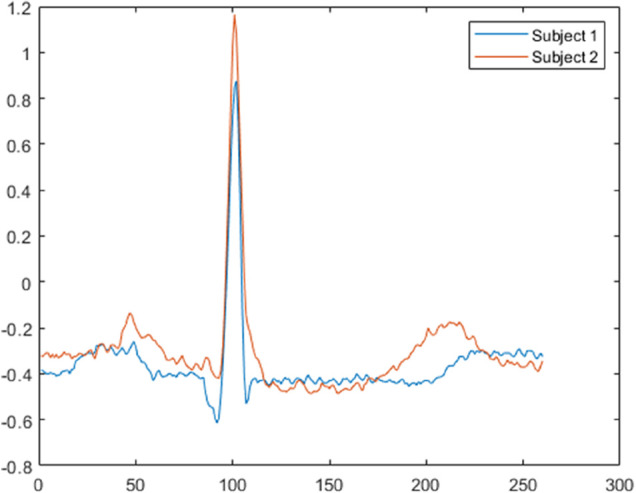
The ECG heartbeats signal of two subjects in MITDB database.

**Fig 3:**
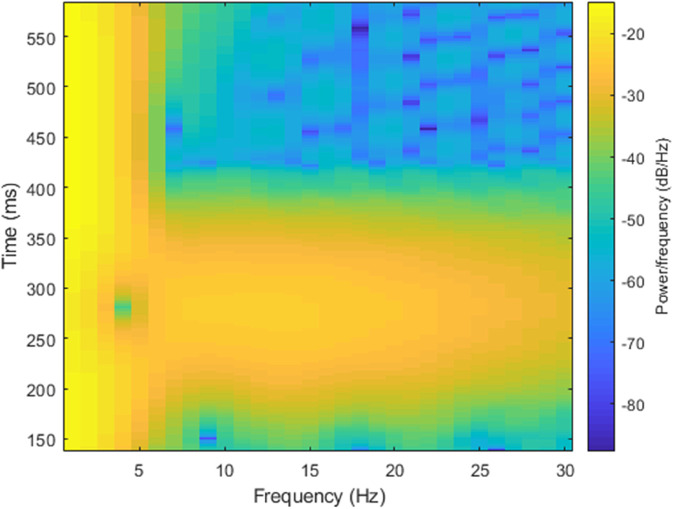
The ECG heartbeat’s spectrogram of subject 1 in MITDB database.

**Fig 4:**
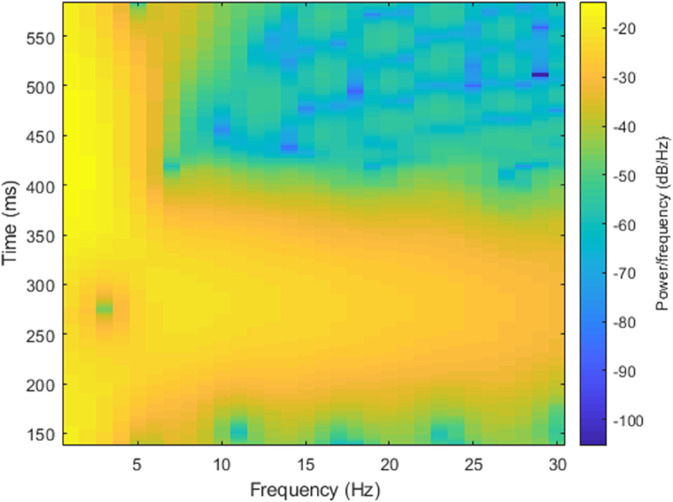
The ECG heartbeat’s Spectrogram of subject 2 in MITDB database.

Due to the non-stationary and nonlinear nature of ECG signals, numerous studies have transformed them into two-dimensional spectrogram images. These spectrograms are able to capture both the temporal and frequecy charactersitics of the signals. Therefore, in this work, we convert ECG heartbeats into spectrograms in accordance with Eq (5).

$$X(R,w)=\int_{-\infty }^{+\infty }y(t)w(t-R)e^{-iwt}dt,$$ (5)

where, *R* is the window function shift, *w* represents the angular frequency. As shown in the Fig 2, two ECG heartbeats from different subjects exhibit noticeable difference. These two signals are converted into spectrograms, which are shown in Figs 3 and 4, respectively. A comparison of these spectrograms reveals distinct variations between them. Consequently, the spectrogram of an ECG signal can be effectively utilized for subject recognition.

### 3.3 Extracting pixel differential vector for spectrogram

**Fig 5:**
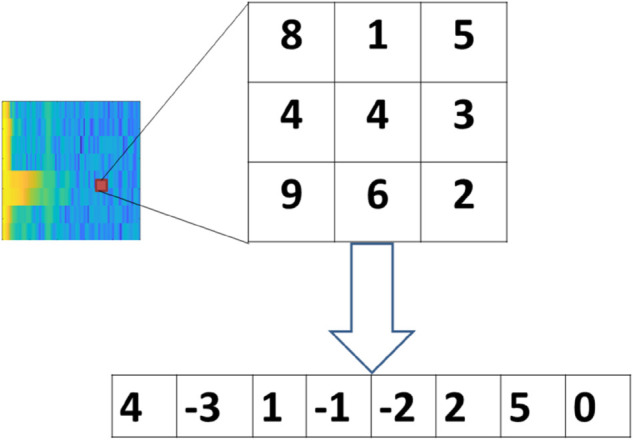
An illustration to show how to extract the PDV from spectrogram of ECG heartbeat.

As is well known, the spectrogram of an ECG heartbeat contains plentiful and distinctive features. Numerous studies have employed deep learning methods to extract effective feature from such spectrogram. However, due to the requirements of large datasets and extensive training time in deep learning, this paper attempts to utilize a bag-of-words framework for the task. The bag-of-words framework necessitates extracting local features from each pixel in the image. While many works adopt hand-crafted features (e.g., LBP and its extensions) as local feature, obtaining optimal features through this appoach remains challenging. In recent years, a variety of feature learning methods have been proposed, including the compact binary face descriptor [16], anchor-based manifold binary pattern [17], and total variation PCA-based descriptors [36]. These studies have manifested that, compared with hand-crafted feature methods, local feature learning can better extract the intrinsic information from data. To learn the local feature, we first construct based feature. As LBP can capture the signs of the difference of the center pixel and neighbors, while it lose the amplitude information of these difference. Therefore, in this paper, we construct a PDV as based feature for our local learning method. An illustration of the PDV operator is presented in Fig 5. For each pixel in the spectrogram, we compute the difference between the pixel and its neighboring pixels in each direction individually, then concatenate these difference vectors to form the PDV. A specific example in Fig 5 to demonstrates how to a PDV are computed from spectrogram. For any given pixel in the spectrogram, we first identity its neighbors within a (2R+1)×(2R+1) spatial window, where *R* is a parameter defining the neighborhood size. For simplicity of illustration, *R* is set to 1 in this figure. Subsequently, the PDV is derived by computing the difference between the center point and each of its neighboring pixels. The size of parameter *R* directly determines the dimensionality of the PDVs. When *R* is 3 and 48 neighboring pixels are selected to form PDV. Although PDV can capture importance information of images, but it is real valued feature and includes redundant information.

### 3.4 Extracting discriminant spectrogram local descriptors

Recently, local feature leaning methods have demonstrated significant potential in face recognition, finger vein recognition, and ECG biometrics. In this work, we aim to design a learning method for constructing a hash function that can transform PDVs into discriminant binary descriptors. Specifically, given a set of training spectrograms from *C* classes, we obtain *n*_*i*_ PDVs from the spectrograms of *i*-th class, forming a set $X_{i}=[x_{i_{1}},...,x_{i_{ni}}]$, i∈1,...,C, and $j\in 1,...,n_{i}$. Here, $x_{ij}\in R^{d}$ presents the *j*-th PDV extracted from the *i*-th class spectrograms, *n*_*i*_ represents the number of PDVs in the *i*-th class, and *d* is the dimension of a PDV. Thus, the overall set of PDVs from all training spectrograms can be expressed as $X=[X_{1},...,X_{C}]\in R^{d\times N}$, where $N=\sum_{i=1}^{C}n_{i}$. Assume that we seek to learn a projection matrix $W=[w_{1},w_{2},...,w_{K}]\in R^{d\times K}$, which maps each *x*_*ij*_ into a binary descriptor $b_{ij}=[b_{1ij},...,b_{Kij}{]}^{T}\in \{0,1{\}}^{K\times 1}$. That is to say, we aim to learn the hashing function as Eq (6).

$$b_{kij}=sgn(W_{k}^{T}x_{ij}),$$ (6)

where *sgn*(*u*) is a sign function, which transforms $u\in R^{K\times 1}$ into a vector in $\{0,1{\}}^{K\times 1}$ based on the sign of each element in *u*. Specifically, *sgn*(*u*) takes the value 1 when *u* > 0 and –1 otherwise.

To ensure each *b*_*ij*_ possesses discriminative power, we introduce several constraints for learning *W*, and the overall objective objective function of DSLD can be formulated as Eq (7). First, we impose a constraint on quantization error to preserve the maximum energy in PDV, which is the first constraint in Eq (7). Second, inspired by critiria of LDA, we enforce the semantic constraints: minimizing the Euclidean distance between intra-class desriptors while maximizing that between the inter-class descriptors. These form the second and third constraints in Eq (7). Third, the final constraint in Eq (7) is an *L*_2,1_ norm constraint to the learned projection matrix, which ensures that the learned binary descriptors exhibit row sparsity to select important features.

$$\min_{W,B}\lambda_{1}||B-W^{T}X|{|}_{F}^{2}+\lambda_{2}||W^{T}S_{W}W|{|}_{F}^{2}-\lambda_{3}||W^{T}S_{b}W|{|}_{F}^{2}+\lambda_{4}||W|{|}_{2,1}\;s.t.\;\;W^{T}W=I,B\in \{0,1{\}}^{K\times N},$$ (7)

where $\lambda_{1},\lambda_{2}$, $\lambda_{3}$ and $\lambda_{4}$ are four positive parameters that balance the contributions of the respective constraint terms. $B=[B_{1},B_{2},...,B_{c}]$ denotes the binary descriptor matrix corresponding to *X*. The *L*_2,1_ norm is defined as $||W|{|}_{2,1}=\sqrt[{2}]{\sum_{j=1}^{d}w_{i,j}^{2}}$, where *w*_*i*,*j*_ represents the element in the *i*-th row and *j*-column of matrix *W*. *S*_*W*_ is the intra-class scatter matrix of PDVs in the training set, here *m*_*i*_ is the mean of all PDVs belonging to *i*-th class across all training spectrograms. *S*_*b*_ is inter-class scatter matrix of PDVs in the training set, and *m* stands for the total mean of all PDVs of the entire training set of spectrograms. From our objective function in Eq (7), the learned descriptors possess the following attributes: (1) Discriminability. The intra-class constraint draws learned descriptors from the same class to be clustered more closely, whereas the inter-class constraint pushes learned descriptors from different classes farther apart. (2) Class structure consistency. The *L*_2,1_ norm constraint endows the learned projection matrix *W* with the row sparsity. When incorporated with the aforementioned intra-class and inter-class constraints, this sparsity property further enhances the discriminative power of the learned descriptors.

### 3.5 Optimization

The objective function in Eq (7) is challenging to optimize due to the non-linear *sgn*(.) function. In this subsection, we perform optimization Eq (7) using an alternate optimization method. The detailed solution steps are presented as follows.

**Step 1. Update *B*:** When *W* are fixed in Eq (7), we can reformulated it as the following equivalent problem:

$$\min_{B}\lambda_{1}||B-W^{T}X|{|}_{F}^{2}\;s.t.\;\;B\in \{0,1{\}}^{K\times N}.$$ (8)

We obtain solution of *B* as:

$$B=sgn(W^{T}X).$$ (9)

**Step 2. Update *W*:** When B is fixed, Eq (7) can be reformulated as the following equivalent problem:

$$\min_{W}\lambda_{1}||B-W^{T}X|{|}_{F}^{2}+\lambda_{2}||W^{T}S_{W}W|{|}_{F}^{2}-\lambda_{3}||W^{T}S_{b}W|{|}_{F}^{2}+\lambda_{4}R||W|{|}_{F}^{2},\;s.t.\;\;W^{T}W=I,$$ (10)

where *R* is a diagonal matrix, and $R_{ii}=\frac{1}{||w_{i}|{|}_{2}}$.

The problem in Eq (10) can be solved using the curvilinear search algorithm proposed in [37] to obtain *W*. We summarize the solving procedure for problem in Eq (7) in Algorithm 1.

**Algorithm 1 DSLD algorithm.**

**Require:**

  *X*= training dataset; ξ= number of iterations; $\lambda_{1}$,$\lambda_{2}$,$\lambda_{3}$ and $\lambda_{4}$= parameters; *K*= length of the descriptor;

**Ensure:**

  Optimized matrix *W*

1: Initialize *W* as the top *K* eigenvectors of *XX^T^*, which correspond to the *K* largest eigenvalues; Initialize *t* as 1;

2: **repeat**

3: Set *t* as *t* + 1;

4: Obtain *B* with fixed W using Eq (9);

5: Learn *W* with fixed B by solving (10);

6: **until** *t*<40 or $|W^{t}-W^{t-1}|<\epsilon$

7: **return** *W*.

### 3.6 ECG heartbeat representation based on DSLD

**Fig 6:**
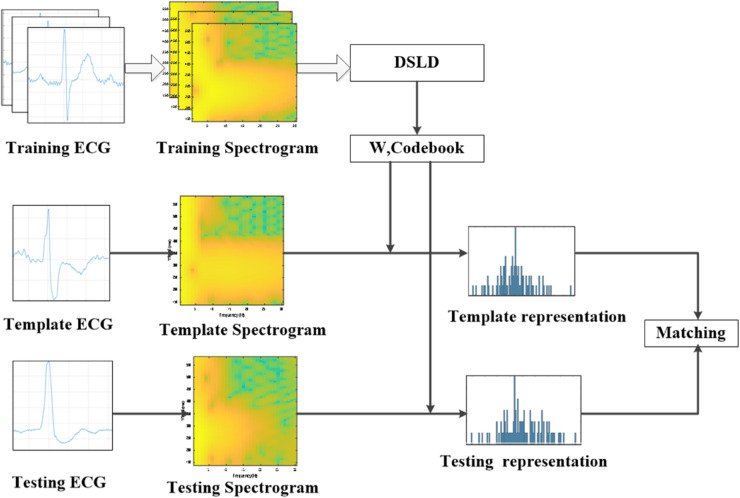
Flowchart of DSLD for ECG representation and authentication.

After obtaining the projective mapping matrix *W*, we generate binary descriptors for all PDVs of the training spectrograms. Then, we cluster these obtained binary descriptors into a codebook using the *k*-means methods. Next, for an ECG heartbeat, we convert it into a spectrogram, extract PDVs from this spectrogram, project these PDVs into binary descriptors by projective mapping matrix *W*, and pool these binary descriptors into the learned codebook to obtain a histogram representation for the ECG heartbeat. The representations of other ECG heartbeat are generated by same manner. Partitioning the image can enhance the performance of the recognition system based on bag-of-words framework. Therefore, we divide the spectrogram into multiple non-overlapping regions and learn the projective mapping matrix and codebook for each region. After obtain the representation of each region for the spectrogram of an ECG heartbeat, we concatenate the histogram representations of all region to form the representation of the ECG heartbeat. The resulting representation of the ECG heartbeat is high-dimensional and includes redundant information. To address this, we employ whitened PCA (WPCA) for dimensionality reduction and calculate the matching score using consine similarity. Fig 6 demonstrates the process of calculating the ECG heartbeat representation using DSLD.

## 4 Experiments and results

We implement the proposed method using MATLAB R2018a and take the experiments on a common desktop PC with i7 3.60 GHZ CPU and 16GB RAM. The MIT-BIH Arrhythmia (MITDB) database is employed to evaluate the performance of the proposed method. To demonstrate its effectiveness, We compare the proposed method with two benchmark methods. The different between the proposed method and the benchmark methods lies in the objective functions employed for learning the local binary descriptors. The objective functions of two benchmark methods are same as PCA and LDA, respectively. Thus, We called them as PCA descriptor (PCAD) and LDA descriptor (LDAD), respectively. Besides, we also compare the proposed method with several state-of-art ECG biometric authentication methods. The performance of the ECG biometric system was evaluated using average recognition accuracy and equal error rate (EER). The average recognition accuracy is defined as the ratio of the number of correctly identified subjects to the total number of subjects in the database. EER refers to the point where the false acceptance rate (FAR) equals the false rejection rate (FRR). The experiments adopted a within-session analysis. In the within-session analysis, both the training set and testing set for a subject come from one recording.

### 4.1 Database and experimental settings

We verified the proposed method using the MIT-BIH Arrhythmia (MITDB) dataset [38]. The MITDB database includes 48 dual-channel recordings from 47 subjects. The frequency of signal in this database is 360 HZ. In our experiments, we first detected the R-peak position using the Pan Tompkin algorithm, then took 100 points forward from the R-peak and 159 points backward in the raw recording to construct an ECG heartbeat. That is to say, 260 points are consisted of one ECG heartbeat. A total 24 heartbeats were selected for each subject. Twelve heartbeats were used to form the training set, and the remaining 12 ECG heartbeats were allocated to the testing set.

### 4.2 Evaluation of DSLD

In this section, we design several experiments to investigate the effectiveness of our objective function by comparing it with two our designed feature learning methods, such as PCAD and LDAD on MITDB database in the within-session. In these experiments, we empirically set the parameters $\lambda_{1}$, $\lambda_{2}$, $\lambda_{3}$ and $\lambda_{4}$ as 1, 1, 0.08, and 10^4^, respectively. Additionally, the codebook size is set as 500, and the length of descriptors is set as 30.

**Fig 7:**
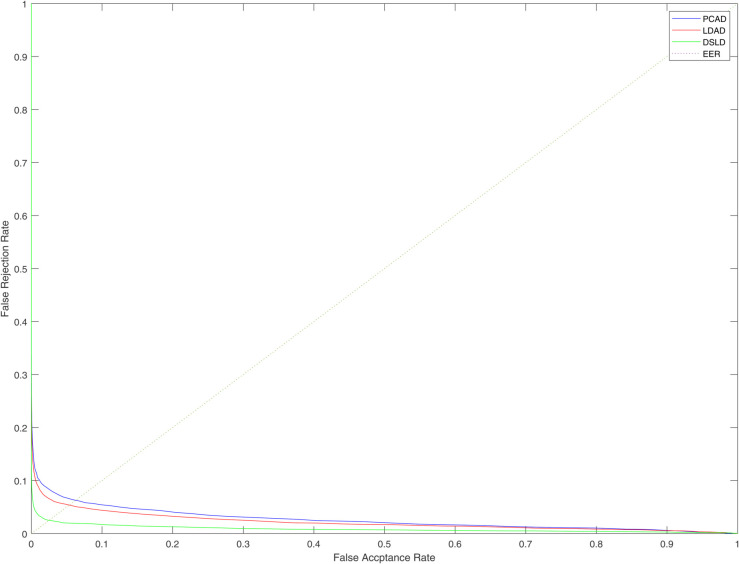
ROC curves of different methods on the MITDB database.

Table 1 illustrates the comparison results, and Fig 7 shows the ROC curves of PCAD, LDAD and DSLD on the MITDB database. We can analyze the results from following aspects. First, the performance of PCAD is compared with that of LDAD. The difference between PCAD and LDAD is the objective function. LDAD enforce the intra-class constraint and inter-class constraint, while PCAD don’t utilize the label information. Table 1 and Fig 7 show that LDAD achieves better performance than PCAD on the MITDB database. The main reason for these results is that LDAD utilize the label information while PCAD do not. Second, the performance of DSLD was compared with that of LDAD. The main difference between them is objective function. DSLD has regulator that is *L*_2,1_ norm of projective matrix, while LDAD has not. Table 1 and Fig 7 demonstrate that DSLD achieves slightly better performance than LDAD. The main reason for these results is that *L*_2,1_ norm make the learned local feature more discrimitive. Therefore, this shows that using intra-class and inter-class constraints and *L*_2,1_ norm of projective matrix can enhance the discrimination of binary descriptor and enhance the recognition performance of ECG biometric system.

**Table 1:** Performace of PCAD, LDAD and DSLD on MITDB database (Average recognition rate and EER).

Methods	Average recognition rate	EER
PCAD	97.89%	0.0613
LDAD	98.23%	0.0546
DSLD	98.60%	0.0241

### 4.3 Comparison with existing ECG biometric authentication methods

In this section we evaluate the performance of our proposed method in comparison with some state-of-art ECG biometric authentication methods [36,39–42]. Table 2 presents the recognition performance of the proposed method compared with the state-of-art methods on the MITDB database. From these results, we can obtain the following conclusions. First, the recognition rate of our method is comparable to or better results than that of the deep learning method in [39] and [42] on the MITDB database.Specifically, the one in [39] relies on a recurrent neural network, while the one in [42] relies on a residual network. These results demonstrate the effectiveness of our proposed method. Second, the recognition rate of DSLD is slightly lower than that of method in [41]. This result lies in the fact that the method in [41] utilizes three heartbeats in their experiment, whereas our only employs one heartbeat. Third, the method in [40] achieves better results than our method on MITDB database. This result comes from that the method [40] used 44 subjects in their experiment, whereas our experiment utilized 47 subjects. Last, our method achieves a better results than the method [36] on the MITDB database. The reason for this lies in the fact that the proposed method transforms the ECG signal into a time-frequency spectrogram, whereas the method in [36] directly utilized the original ECG signal.

**Table 2:** Comparison with different conventional biometric authentication methods on MITDB database.

Methods	Databases (Subject number)	Number of heartbeats	Accuracy
[39]	MITDB(47)	1	98.60%
[40]	MITDB(44)	1	98.80%
[41]	MITDB(47)	3	100%
[42]	MITDB(47)	1	95.99%
[36]	MITDB(47)	1	98.31%
DSLD	MITDB(47)	1	98.60%

**Table 3:** Training time and matching time per heartbeat.

Databases	Training time (s)	Matching time per heartbeat (ms)
MITDB	859.709s	36.8ms

In addition, to further assess the feasibility of our method in real-world application scenarios, we recorded both the training time and matching time. The training time includes the conversion time from ECG heartbeat to spectrogram, the learning time of the project map matrix during the training phase, and the computation time for the template representation. The matching time refers to the duration from the input of test ECG heartbeats to the generation of the authentication result. As shown in Table 3, the matching time is shorter than the training time on the MITDB database, and the matching time is extremely low. Therefore, our proposed method is well-suited for practical application.

## 5 Conclusions

This paper proposes a novel local binary feature learning method for ECG heartbeat spectrograms, called discriminant spectrogram local descriptors(DSLD) for ECG biometric authentication. Unlike traditional feature descriptors that directly extract features from the original ECG heartbeat, DSLD obtains local features from the spectrogram of ECG heartbeat. In constrast to hand-designed feature descriptors that require domain knowledge of ECG, DSLD simply extracts PDVs from ECG heartbeat spectrogram and learns discriminative descriptor by incorporating with LDA criterion and the *L*_2,1_ norm. In addition, in the feature learning process of DSLD, we construct an objective function that simultaneously considers quantization loss, label semantic information between the inter-class and intra-class samples, as well as the sparsity property. Therefore, the descriptors extracting by DSLD is different from other binary feature learning methods. Moreover, we utilize the bag-of-words framework organize the learned descriptors for representating the ECG heartbeat. Experimental results on publicly accessible ECG database show that DSLD consistently achieved a better performance than the state-of-the-art ECG identity recognition methods.

## References

[1] Biel L, Pettersson O, Philipson L, Wide P. ECG analysis: a new approach in human identification. IEEE Trans Instrum Meas. 2001;50(3):808–12. doi: 10.1109/19.930458

[2] Paiva JS, Dias D, Cunha JPS. Beat-ID: Towards a computationally low-cost single heartbeat biometric identity check system based on electrocardiogram wave morphology. PLoS One. 2017;12(7):e0180942. doi: 10.1371/journal.pone.0180942 28719614 PMC5515426

[3] Choi H-S, Lee B, Yoon S. Biometric authentication using noisy electrocardiograms acquired by mobile sensors. IEEE Access. 2016;4:1266–73. doi: 10.1109/access.2016.2548519

[4] Dar MN, Akram MU, Usman A, Khan SA. ECG biometric identification for general population using multiresolution analysis of DWT based features. In: 2015 Second International Conference on Information Security and Cyber Forensics (InfoSec). 2015. p. 5–10. 10.1109/infosec.2015.7435498

[5] Xu J, Yang G, Wang K, Huang Y, Liu H, Yin Y. Structural sparse representation with class-specific dictionary for ECG biometric recognition. Pattern Recognition Letters. 2020;135:44–9. doi: 10.1016/j.patrec.2020.04.022

[6] Biran A, Jeremic A. ECG bio-identification using Fréchet classifiers: a proposed methodology based on modeling the dynamic change of the ECG features. Biomedical Signal Processing and Control. 2023;82:104575. doi: 10.1016/j.bspc.2023.104575

[7] Benouis M, Mostefai L, Costen N, Regouid M. ECG based biometric identification using one-dimensional local difference pattern. Biomedical Signal Processing and Control. 2021;64:102226. doi: 10.1016/j.bspc.2020.102226

[8] da Silva Luz EJ, Moreira GJP, Oliveira LS, Schwartz WR, Menotti D. Learning deep off-the-person heart biometrics representations. IEEE TransInformForensic Secur. 2018;13(5):1258–70. doi: 10.1109/tifs.2017.2784362

[9] Donida Labati R, Muñoz E, Piuri V, Sassi R, Scotti F. Deep-ECG: convolutional neural networks for ECG biometric recognition. Pattern Recognition Letters. 2019;126:78–85. doi: 10.1016/j.patrec.2018.03.028

[10] Zhao Z, Zhang Y, Deng Y, Zhang X. ECG authentication system design incorporating a convolutional neural network and generalized S-Transformation. Comput Biol Med. 2018;102:168–79. doi: 10.1016/j.compbiomed.2018.09.027 30290297

[11] Meltzer D, Luengo D. Efficient clustering-based electrocardiographic biometric identification. Expert Systems with Applications. 2023;219:119609. doi: 10.1016/j.eswa.2023.119609

[12] Song CH, Kim JS, Kim JM, Pan S. Stress classification using ECGs based on a multi-dimensional feature fusion of LSTM and Xception. IEEE Access. 2024;12:19077–86. doi: 10.1109/access.2024.3361684

[13] Chen J, Fang B, Li H, Zhang L-B, Teng Y, Fortino G. EMCNet: ensemble multiscale convolutional neural network for single-lead ECG classification in wearable devices. IEEE Sensors J. 2024;24(6):8754–62. doi: 10.1109/jsen.2024.3358997

[14] Li Y, Pang Y, Wang K, Li X. Toward improving ECG biometric identification using cascaded convolutional neural networks. Neurocomputing. 2020;391:83–95. doi: 10.1016/j.neucom.2020.01.019

[15] Zhu G, Ma M, Huang Y, Wang K, Yang G. Dual-domain low-rank fusion deep metric learning for off-the-person ECG biometrics. In: ICASSP 2022 - 2022 IEEE International Conference on Acoustics, Speech and Signal Processing (ICASSP). 2022. p. 2914–8. 10.1109/icassp43922.2022.9747122

[16] Lu J, Liong VE, Zhou X, Zhou J. Learning compact binary face descriptor for face recognition. IEEE Trans Pattern Anal Mach Intell. 2015;37(10):2041–56. doi: 10.1109/TPAMI.2015.2408359 26340256

[17] Liu H, Yang G, Yang L, Su K, Yin Y. Anchor-based manifold binary pattern for finger vein recognition. Sci China Inf Sci. 2019;62(5). doi: 10.1007/s11432-018-9651-8

[18] Ma S, Hu Q, Zhao S, Wu W, Wu J. Multiscale multidirection binary pattern learning for discriminant palmprint identification. IEEE Trans Instrum Meas. 2023;72:1–12. doi: 10.1109/tim.2023.323875337323850

[19] Zha Z, Tang H, Sun Y, Tang J. Boosting few-shot fine-grained recognition with background suppression and foreground alignment. IEEE Trans Circuits Syst Video Technol. 2023;33(8):3947–61. doi: 10.1109/tcsvt.2023.3236636

[20] Yan S, Tang H, Zhang L, Tang J. Image-specific information suppression and implicit local alignment for text-based person search. IEEE Trans Neural Netw Learn Syst. 2024;35(12):17973–86. doi: 10.1109/TNNLS.2023.3310118 37713222

[21] Palaniappan R, Krishnan SM. Identifying individuals using ECG beats. In: 2004 International Conference on Signal Processing and Communications 2004 . SPCOM ’04. p. 569–72. 10.1109/spcom.2004.1458524

[22] Srivastva R, Singh YN. ECG analysis for human recognition using non-fiducial methods. IET Biometrics. 2019;8(5):295–305. doi: 10.1049/iet-bmt.2018.5093

[23] Gutta S, Cheng Q. Joint feature extraction and classifier design for ECG-based biometric recognition. IEEE J Biomed Health Inform. 2016;20(2):460–8. doi: 10.1109/JBHI.2015.2402199 25680220

[24] Wang K, Yang G, Huang Y, Yin Y. Multi-scale differential feature for ECG biometrics with collective matrix factorization. Pattern Recognition. 2020;102:107211. doi: 10.1016/j.patcog.2020.107211

[25] Huang Y, Yang G, Wang K, Liu H, Yin Y. Learning joint and specific patterns: a unified sparse representation for off-the-person ECG biometric recognition. IEEE TransInformForensic Secur. 2021;16:147–60. doi: 10.1109/tifs.2020.3006384

[26] Fatimah B, Singh P, Singhal A, Pachori RB. Biometric identification from ECG signals using fourier decomposition and machine learning. IEEE Trans Instrum Meas. 2022;71:1–9. doi: 10.1109/tim.2022.3199260

[27] Abdeldayem SS, Bourlai T. A novel approach for ECG-based human identification using spectral correlation and deep learning. IEEE Trans Biom Behav Identity Sci. 2020;2(1):1–14. doi: 10.1109/tbiom.2019.2947434

[28] Salloum R, Kuo C-CJ. ECG-based biometrics using recurrent neural networks. In: 2017 IEEE International Conference on Acoustics, Speech and Signal Processing (ICASSP). 2017. p. 2062–6. 10.1109/icassp.2017.7952519

[29] Byeon Y-H, Pan S-B, Kwak K-C. Ensemble deep learning models for ECG-based biometrics. In: 2020 Cybernetics & Informatics (K&I). 2020. p. 1–5. 10.1109/ki48306.2020.9039871

[30] Srivastva R, Singh A, Singh YN. PlexNet: a fast and robust ECG biometric system for human recognition. Information Sciences. 2021;558:208–28. doi: 10.1016/j.ins.2021.01.001

[31] Thentu S, Cordeiro R, Park Y, Karimian N. ECG biometric using 2D deep convolutional neural network. In: 2021 IEEE International Conference on Consumer Electronics (ICCE). 2021. p. 1–6. 10.1109/icce50685.2021.9427616

[32] Kim B-H, Pyun J-Y. ECG identification for personal authentication using LSTM-based deep recurrent neural networks. Sensors (Basel). 2020;20(11):3069. doi: 10.3390/s20113069 32485827 PMC7309053

[33] Fuster-Barceló C, Camara C, Peris-López P. Unleashing the power of electrocardiograms: a novel approach for patient identification in healthcare systems with ECG signals; arXiv prerprint 2023. http://arxiv.org/abs/2302.06529

[34] Jyotishi D, Dandapat S. An ECG biometric system using hierarchical LSTM with attention mechanism. IEEE Sensors J. 2022;22(6):6052–61. doi: 10.1109/jsen.2021.3139135

[35] Islam MS, Elwarfalli I. Deep learning-powered ECG-based biometric authentication. In: 2023 International Conference on Next-Generation Computing, IoT and Machine Learning (NCIM). 2023. p. 1–6. 10.1109/ncim59001.2023.10212845

[36] Liu H, Lin H, Wang X. Total variation PCA-based descriptors for electrocardiography identity recognition. IEEE Access. 2024;12:3815–24. doi: 10.1109/access.2023.3349148

[37] Wen Z, Yin W. A feasible method for optimization with orthogonality constraints. Math Program. 2012;142(1–2):397–434. doi: 10.1007/s10107-012-0584-1

[38] Moody GB, Mark RG. The impact of the MIT-BIH arrhythmia database. IEEE Eng Med Biol Mag. 2001;20(3):45–50. doi: 10.1109/51.932724 11446209

[39] Salloum R, Kuo C-CJ. ECG-based biometrics using recurrent neural networks. In: 2017 IEEE International Conference on Acoustics, Speech and Signal Processing (ICASSP). 2017. p. 2062–6. 10.1109/icassp.2017.7952519

[40] Li R, Yang G, Wang K, Huang Y, Yuan F, Yin Y. Robust ECG biometrics using GNMF and sparse representation. Pattern Recognition Letters. 2020;129:70–6. doi: 10.1016/j.patrec.2019.11.005

[41] Li R, Yang G, Wang K, Huang Y, Yuan F, Yin Y. Robust ECG biometrics using GNMF and sparse representation. Pattern Recognition Letters. 2020;129:70–6. doi: 10.1016/j.patrec.2019.11.005

[42] Chu Y, Shen H, Huang K. ECG authentication method based on parallel multi-scale one-dimensional residual network with center and margin loss. IEEE Access. 2019;7:51598–607. doi: 10.1109/access.2019.2912519

